# Mesenchymal stem cells from different sources for sepsis treatment: prospects and limitations

**DOI:** 10.1590/1414-431X2024e13457

**Published:** 2024-10-14

**Authors:** Heng Chen, Xiaosui Ling, Bo Zhao, Jing Chen, XianYi Sun, Jing Yang, Pibao Li

**Affiliations:** 1 Shandong University of Traditional Chinese Medicine, Jinan, Shandong, China; 2 The Sixth Affiliated Hospital, Sun Yat-Sen University, Guangzhou, Guangdong, China; 3 Department of Intensive Care Unit, The First Rehabilitation Hospital of Shandong, Linyi, Shandong, China; 4 Department of Forensic Medicine, Yuancheng District Public Security Bureau, Heyuan, Guangdong, China; 5 Department of Pharmacy, Shandong Provincial Third Hospital, Cheeloo College of Medicine, Shandong University, Jinan, Shandong, China; 6 Department of Pharmacy, Shandong Medical College, Jinan, Shandong, China

**Keywords:** Mesenchymal stem cells, Sepsis, MSC therapies, Clinical trials, Comparison

## Abstract

Sepsis is a systemic inflammatory response syndrome in which the host response to infection is dysregulated, leading to circulatory dysfunction and multi-organ damage. It has a high mortality rate and its incidence is increasing year by year, posing a serious threat to human life and health. Mesenchymal stem cells (MSC) have the following properties: hematopoietic support, provision of nutrients, activation of endogenous stem/progenitor cells, repair of tissue damage, elimination of inflammation, immunomodulation, promotion of neovascularization, chemotaxis and migration, anti-apoptosis, anti-oxidation, anti-fibrosis, homing, and many other effects. A large number of studies have confirmed that MSC from different sources have their own characteristics. This article reviews the pathogenesis of sepsis, the biological properties of MSC, and the advantages and disadvantages of different sources of MSC for the treatment of sepsis and their characteristics.

## Introduction

Sepsis is a systemic inflammatory response syndrome in which the host response to infection is dysregulated leading to circulatory dysfunction and multi-organ damage. According to international epidemiological surveys, there are approximately 31.5 million cases of sepsis and 19.4 million cases of severe sepsis globally each year, and approximately 5.3 million deaths due to sepsis each year (1). In recent years, although great progress has been made in the treatment of sepsis, the mortality rate of septic patients is still as high as 25-30%, and the mortality rate of patients with septic shock is even higher than 50% (2-4). According to the latest statistics, 1.7 million adults are affected by sepsis and nearly 270,000 people die from sepsis in the United States each year (5). Its high mortality rate and increasing morbidity rate year by year seriously threaten human life and health (6). Despite the continuous development of critical care medicine in recent years, which has led to greater progress in the treatment of critical illnesses, the conventional treatment of sepsis is not effective, and there is an urgent need to explore new treatment methods.

Mesenchymal stem cells (MSC) show great promise as a treatment for sepsis due to their unique properties. Research on the mechanisms behind sepsis progression indicates that MSC may be able to counteract some of the damaging effects. MSC can be obtained from various tissue sources, each with advantages and disadvantages. Moreover, research experiments have proven that MSC have achieved good results in the treatment of sepsis and are expected to become a new candidate for the treatment of sepsis (7). In addition, different sources of MSC have their own advantages, disadvantages, and characteristics, mainly reflecting differences in their content, proliferation ability, immunomodulatory ability, and secretion of cytokine. Therefore, this paper makes a side-by-side comparison of the advantages, disadvantages, and characteristics of different sources of MSC for sepsis treatment.

## The pathogenetic process of sepsis

**Figure 1:**
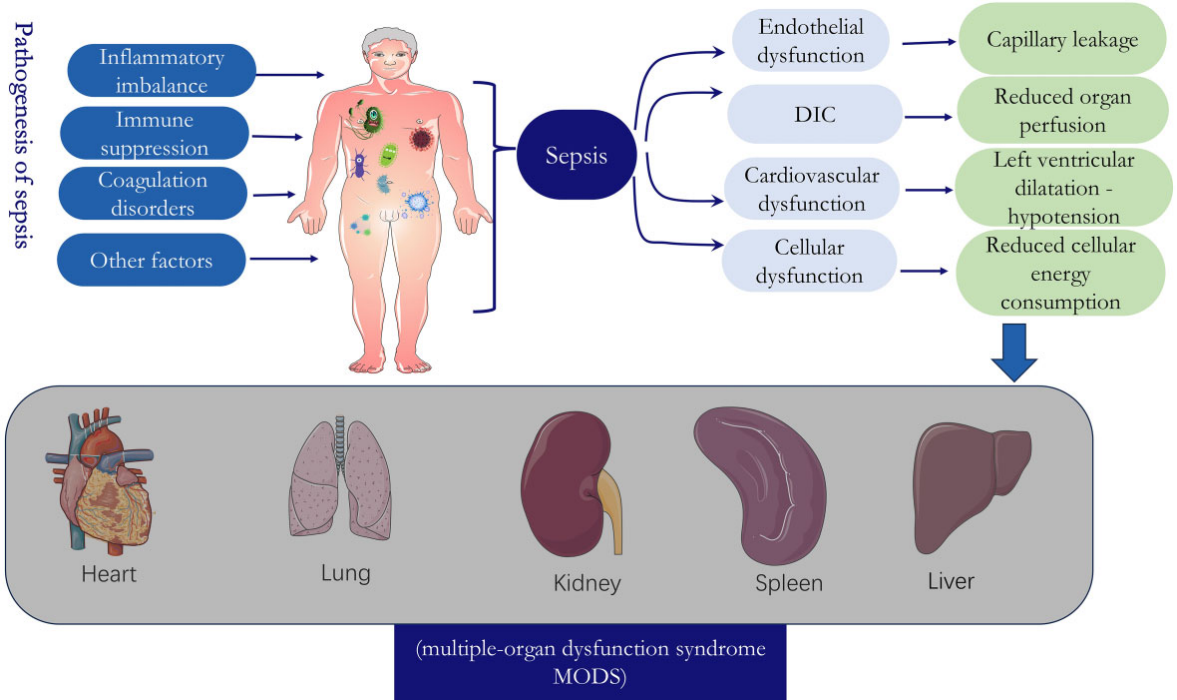
Physiologic and pathologic changes and adverse outcomes during sepsis. The pathogenesis of sepsis includes 1) inflammatory imbalance; 2) immunosuppression; 3) coagulation disorders; and 4) other factors. These factors can cause vascular endothelial dysfunction, disseminated intravascular coagulation (DIC), cardiovascular dysfunction, and cellular dysfunction. Further conditions include capillary leakage, reduced organ perfusion, left ventricular dilatational hypotension, and reduced cellular energy consumption, which can ultimately lead to the development of multiple organ dysfunction syndrome (MODS).

The pathogenesis of sepsis is very complex, involving functional changes in multiple organs of the body, which can induce septic shock and multiple-organ dysfunction syndrome (MODS). In the past, it was believed that sepsis was mainly due to tissue and organ damage caused by excessive inflammatory response, but more and more studies have found that the pathophysiological process of sepsis is very complex, including multiple aspects of inflammation, immunity, and coagulation dysfunction, and involves a variety of alterations in cell function, metabolism, and microcirculation (8). First and foremost, inflammatory imbalance is the most important foundation in the pathogenesis of sepsis and is present throughout the course of sepsis. Pathogens that elicit an inflammatory response include bacteria, fungi, parasites, and viruses. The initial acute response of the host to an invasive pathogen typically involves the release of inflammatory mediators, triggering an inflammatory reaction. This release attracts immune cells such as macrophages, dendritic cells, and T cells to phagocytose and clear the pathogen (9). Second, if inflammation is not effectively controlled, inflammatory mediators will continue to increase, anti-inflammatory responses will be enhanced, and the body will gradually enter a state of immune paralysis/immunosuppression. There are many mechanisms by which sepsis induces immunosuppression, including depletion of immune cells by apoptosis, increase in T regulatory cells and myeloid-derived suppressor cells, and cellular exhaustion (10). Third, under normal conditions, coagulation activation is regulated by three key physiological anticoagulation pathway systems, including the tissue factor pathway inhibitory system, the activated protein C (APC) system, and the antithrombotic system, which regulate coagulation activation, whereas during sepsis, all three pathways are disturbed to some extent (11). Fourth, other factors that influence the development of sepsis include neuro-endocrine-immune networks, cellular autophagy, mitochondrial damage, endoplasmic reticulum stress, and genetic polymorphisms (12) (Figure 1).

## Biological properties of MSC

**Figure 2:**
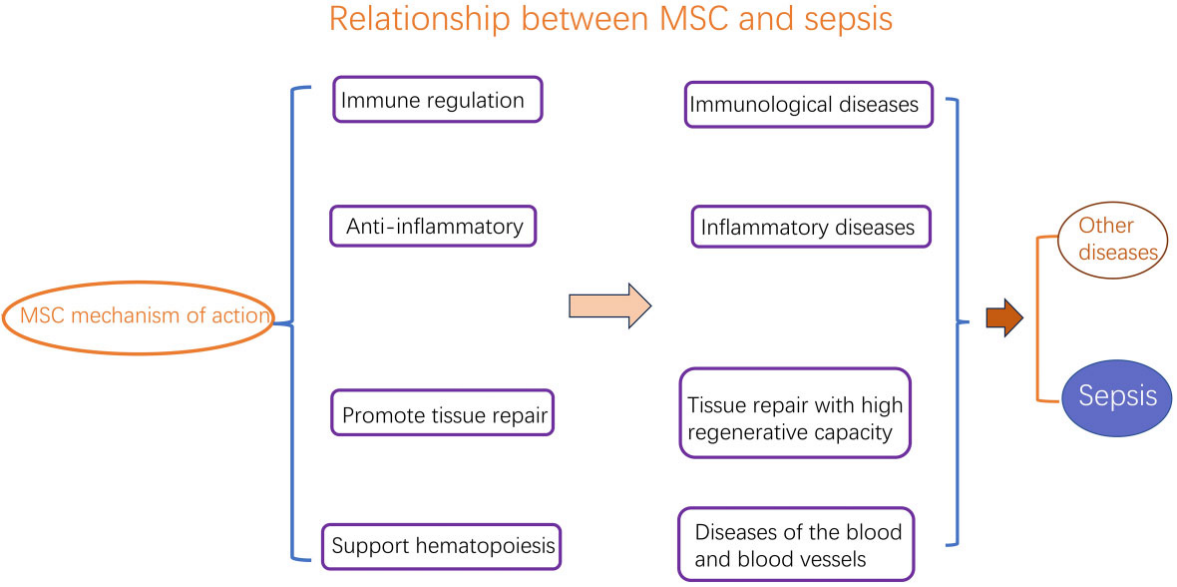
Relationship between mesenchymal stem cells (MSC) biological characteristics and disease. MSC has mainly immunomodulatory, anti-inflammatory, tissue repair with high regenerative capacity, and hematopoietic-supporting biological properties, with potential therapeutic effects on immunological and inflammatory diseases, tissue repair, and blood and vascular-related diseases.

MSC is a class of pluripotent adult stem cells with stem cell characteristics isolated and cultured from the mesoderm and ectoderm of various tissues and organs. MSC is an important class of cell type in the family of stem cells and is the most representative adult stem cell with excellent biological properties such as wide source, strong multidirectional differentiation ability, high plasticity, low immunogenicity, paracrine secretion of various bioactive factors, and low risk of teratogenesis and tumorigenicity (13). MSC have a variety of effects including hematopoietic support, provision of nutrients, activation of endogenous stem/progenitor cells, tissue damage repair, elimination of inflammation, immunomodulation, promotion of neovascularization, chemotaxis and migration, anti-apoptosis, antioxidant, anti-fibrotic, homing, and so on (Figure 2).

## MSC from different sources

MSC are widely found in bone marrow, umbilical cord blood, umbilical cord, placenta, adipose tissue, and other tissues. Current research has shown that the characteristics of MSC from different tissue sources in the treatment of sepsis are different. In this paper, we review the research progress related to the treatment of sepsis with BMSC, ADSC, UC-MSC, UCB-MSC, and MenSC, with the aim of providing reference for the best application of MSC from different sources in the treatment of sepsis.

### Bone marrow mesenchymal stem cells (BMSC)

In 1986, Friedenstein et al. first discovered non-hematopoietic pluripotent cells in the bone marrow mesenchyme, which was the first type of mesenchymal stem cell discovered. These were later named bone marrow mesenchymal stem cells (BMSC) and were found to have a strong medical value (14). With the continuous development of stem cell technology, the effectiveness of BMSC in the treatment of sepsis has received widespread attention.

Basic studies on BMSC for sepsis are abundant. First, it has been shown that BMSC can block the PI3K/AKT/mTOR signaling pathway and reverse endotoxemia and high expression of inflammatory cytokines in rats with acute liver failure (15). Selim et al. (16) found that BMSC could counteract oxidative damage and treat sepsis-induced liver injury by activating Nrf2 signaling. In addition, researchers have also found that nuclear factor-red lineage 2p45-related factor 2 (Nrf2)-mediated heme oxygenase-1 (HO-1) signaling pathway plays a key role in the protective effect against sepsis-induced acute lung injury (17). Secondly, BMSC can treat sepsis through an immunomodulatory mechanism. Han et al. (18) found that synthetic glycan-2 (SDC2) expressed by BMSC could modulate the immune response during sepsis and promote bacterial clearance and inflammatory regression. In addition, anti-inflammatory factors secreted by BMSC exosomes could also attenuate systemic sepsis response. In an experiment to study the protective effect and mechanism of BMSC on lung injury in *Vibrio traumaticus* sepsis, it was found that the expression levels of tumor necrosis factor (TNF)-α, interleukin (IL)-1β, IL-6, and nuclear factor (NF)-κBp65 in the nuclei of lung tissues of *V. traumaticus*-infected mice were significantly increased, and the expression levels were significantly decreased after BMSC intervention, suggesting that anti-inflammatory factors secreted by BMSC exosomes have a therapeutic effect on the lung injury induced by *V. traumaticus* sepsis (19). In addition, another study found that BMSC secrete a higher amount of VEGF (vascular endothelial growth factor) than umbilical cord mesenchymal stem cells (UC-MSC) (20). This suggests that BMSC may have a better repairing effect on blood and vascular damage in sepsis and can improve tissue ischemia and hypoxia.

McIntyre et al. (21) conducted a dose-escalation trial of BMSC for treating infectious shock. They recruited nine participants within 24 h of intensive care unit admission and demonstrated the safety of infusing freshly cultured allogeneic BMSC at doses up to 3 million cells per kilogram (250 million cells).

BMSC treatment of sepsis has its own advantages and limitations, and it has been shown that BMSC express significantly higher levels of sVEGFR1 and sTNFR1, two soluble cytokine receptors with known therapeutic activity in sepsis, compared to adipose derived stem cells (ADSC). Therefore, BMSC may be more effective as a cell therapy for the treatment of endotoxic shock (22). However, the inflammatory environment can increase the expression of human leucocyte antigen (HLA)-DR in BMSC (about 12%) (23). The increase of HLA-DR means that the immunogenicity of MSC is increased, and the antigen-presenting cells of the immune system recognize the MSC with high expression of HLA-DR and present them to T cells for killing and removal. The high expression of HLA-DR leads to the acceleration of the body's removal of MSC and the shortening of the time for MSC to play a role in the body, which directly affects the therapeutic effect. However, the inflammatory environment cannot increase the expression of HLA-DR in UC-MSC and umbilical cord blood mesenchymal stem cells (UCB-MSC) (24).

In summary, the therapeutic effects of BMSC on sepsis involve multiple interacting mechanisms. Key areas of research are BMSC through the regulation of signaling pathways/molecules associated with inflammation, immunomodulation, and exosomes to secrete anti-inflammatory factors. Clinical trials have proven that BMSC infusion is safe for septic patients. BMSC may be more effective as a cellular therapy for the treatment of septic shock when compared to ADSC. BMSC have a better ability to improve tissue hypoxia and ischemia than UC-MSC. However, in the inflammatory environment, BMSC have less time to act than UC-MSC and USB-MSC.

Although BMSC have great therapeutic potential for sepsis, their use might be hampered due to a high level of viral infection, significant decline in cell number and ability to proliferate and differentiate with age, and its invasive nature (25).

### Adipose derived stem cells (ADSC)

Adipose-derived mesenchymal stem cells (ADSC) are an important component of MSC. ADSC are MSC characterized by a multipotent spectrum. They are readily available, and the proportion of stem cells in adipose tissue lysate is the highest of all other MSC sources. They can be obtained by simple, rapid, and noninvasive liposuction of the abdomen, flanks, and/or thighs using a 3-millimeter (mm)-diameter cannula (26).

Shen et al. (27) found that ADSC exosomes could ameliorate the inflammatory response and injury in multiorgan sepsis by polarizing macrophages to an anti-inflammatory phenotype through regulating the expression of Nrf2 and HO-1. *In vivo* experimental studies also found that ADSC exosomes could attenuate sepsis-induced lung injury in mice by inhibiting IL-27 secretion from macrophages (28). Differently, it was found (29) that, when comparing the different lung protective effects of exosomes derived from ADSC, BMSC, and UC-MSC on sepsis-induced acute lung injury, ADSC-derived exosomes were superior to the BMSC- and UC-MSC-derived exosomes. Secondly, in terms of immune mechanisms, ADSC are considered to have a stronger immunomodulatory effect than BMSC. It has been reported that ADSC are less sensitive to natural killer cell-mediated lysis than BMSC, and thus may remain in tissues long enough to balance the immune response before being cleared (30).

In a clinical trial on ADSC treatment of sepsis, Alp et al. (31) found that the survival rate of patients with sepsis and septic shock on days 14 and 28 was higher in the ADSC infusion group than in the control group, and the early mortality rate in the control group was higher than that in the ADSC infusion group, which may indicate that ADSC treatment has a positive effect on survival in early sepsis.

In summary, ADSC can be used to treat sepsis via exosomes to alter macrophage polarization phenotype, secretion of cytokines, and immunomodulation. In clinical trials, ADSC treatment had a positive impact on survival in early sepsis. ADSC have a stronger immunomodulatory effect than BMSC. Moreover, for the treatment of sepsis, exosomes of ADSC may be superior to BMSC and UC-MSC. ADSC have more than one thousand times higher cell yield, as well as longer lifespan, higher proliferation capacity, and shorter propagation time (26). In addition, ADSC have the ability to regulate inflammation and ameliorate ischemia-reperfusion injury (32). Although ADSC have many advantages over BMSC, there are still many unsolved problems in their use in the treatment of sepsis, such as the different differentiation ability of ADSC originating from different individuals and different sites and the challenge of selecting the clinical treatment from disease to disease, the combination with other treatment modalities, the clinical dosage, the time of application, and so on.

### Umbilical cord mesenchymal stem cells (UC-MSC)

The umbilical cord consists of three parts: umbilical vessels, amniotic epithelium, and Wharton's gel (33). The umbilical cord is an extraembryonic tissue that contains a large number of differentiated stem cells. In recent years, several researchers have isolated and obtained UC-MSC from different structures, such as the whole umbilical cord or part of the tissue, and used them for the treatment of sepsis, and UC-MSC have received widespread attention.

Studies have reported that UC-MSC can secrete anti-inflammatory factors through exosomes to alleviate the sepsis response. A mouse model study by Zhang et al. (34) found that UC-MSC exosomes ultimately alleviated sepsis-associated acute kidney injury (AKI) by regulating the expression of microRNA-146b, which led to the inhibition of NF-κB activity. Animal experiments by Zhang et al. (35) demonstrated that UC-MSC attenuated inflammation, apoptosis and neuronal damage, and cognitive dysfunction in an animal model of sepsis-associated encephalopathy through a PI3K/AKT-dependent pathway, which resulted in a reduction in the secretion of IL-6, IL-1β, and HMGB1, as well as the activation of NF-κB. It has been shown that a greater number of cytokines are secreted by UC-MSC (G-CSF, GM-CSF, HGF, IL-6, IL-8, IL-11) than by BMSC (36).

UC-MSC for the treatment of sepsis have been studied in relatively more clinical trials and shown to be safer and more reliable. A phase I clinical trial of UC-MSC for the treatment of severe sepsis conducted by He et al. (37) demonstrated that treatment with UC-MSC at doses up to 3×10^6^ cells/kg was safe and well tolerated in 15 patients with severe sepsis. In global outbreaks, patients with severe COVID-19 usually develop acute respiratory distress syndrome, sepsis, uncorrectable metabolic acidosis, coagulation disorders, multi-organ failure, and even death within a short period of time after onset. Rebelatto et al. (38). conducted a clinical trial on 17 COVID-19 patients with severe infections. The patients were treated with UC-MSC and compared with a control group. On day 14, the UC-MSC group showed significant decreases in serum ferritin, IL-6, and MCP1-CCL2 compared to control. After 2 months of treatment, the UC-MSC group also had decreased C-reactive protein and neutrophil levels. These data suggest that early infusion of UC-MSC in critically ill patients with COVID-19 is safe and can play an important role in both preventing serious complications and reducing the chronic phase of acute sequelae. In addition, UC-MSC have an impact on the prognosis of COVID-19 critically ill patients. The clinical trial by Meng et al. (39) included 40 COVID-19 critically ill patients, 20 randomly assigned to be treated with UC-MSC and 20 patients served as a control group. All of them were treated with standard therapy, and it was found that the survival rate of the UC-MSC group was 2.5 times higher.

In summary, UC-MSC can attenuate sepsis through exosomal secretion of relevant anti-inflammatory factors and modulation of inflammation-related pathways. In clinical trials, UC-MSC play an important role in preventing complications and reducing acute sequelae and have a positive impact on survival in patients with severe infections. Moreover, some of the anti-inflammatory factors secreted by UC-MSC were higher than those secreted by BMSC. Since MSC are age-specific, there is a significant decrease in the number and proliferative capacity of MSC with age. Therefore, the proliferation ability of MSC from tissue sources such as umbilical cord, umbilical cord blood, amniotic membrane, amniotic fluid, and placenta is stronger than that of adult tissues (including BMSC, ADSC, etc.). Compared with BMSC and ADSC, UC-MSC are used more often in clinical trials due to their unique characteristics, including 1) high MSC content in umbilical cord, high proliferative capacity, abundant sources, and easy collection, storage, and transport; 2) less ethical controversy; 3) pluripotency, which allows them to differentiate into a variety of cell types; and 4) low immunogenicity with a immunosuppressive ability (40). Therefore, human umbilical cord tissue can be considered as the most promising source of MSC.

### Umbilical cord blood mesenchymal stem cells (UCB-MSC)

Umbilical cord blood is the blood remaining in the placenta and umbilical cord after delivery of the fetus, ligation, and disconnection of the umbilical cord; the number of MSC in umbilical cord blood is very small. In 2000, Erices et al. (41) reported for the first time the isolation and culture of UCB-MSC.

Recently, it has been shown that UCB-MSC can be used to treat sepsis by binding to neutrophils. Ahn et al. (42) investigated the interaction between mouse liver neutrophils and UCB-MSC during sepsis and found that human-derived UCB-MSC were not recognized as a foreign body in mice and did not cause graft rejection, and that UCB-MSC had a beneficial effect on LPS-induced sepsis through its binding to neutrophils.

In summary, UCB-MSC can treat sepsis by binding to neutrophils in animal studies. It has been reported that the immunomodulatory ability of UC-MSC is significantly better than that of BMSC and UCB-MSC, while UCB-MSC have the worst immunosuppressive ability (43). However, UCB-MSC also have their own advantages; UCB-MSC secrete more cytokines that support hematopoiesis and therefore support hematopoietic stem cell clone formation better than BMSC (44). At present, regarding UCB-MSC in the process of *in vitro* induction culture, there are problems such as low culture yield, difficult culture, long culture time, and difficult storage, which make it difficult to bear the burden of treatment, and it is still necessary to continue to explore how to overcome these difficulties.

### Menstrual blood derived mesenchymal stem cells (MenSC)

In 2007, for the first time in humans, a new source of stem cells was identified from menstruation, and these cells were subsequently named menstrual blood mesenchymal stem cells (45). Menstruation results from the shedding of the endometrium, which is highly regenerative. The functional layer is shed during menstruation and then regenerated from the basal layer during the subsequent menstrual cycle.

There are few studies and experiments on MenSC, but they open up new treatment options. Alcayaga-Miranda et al. (46) found a synergistic effect of MenSC in combination with antibiotics in a mouse model of cecum ligation and puncture sepsis, which may improve the survival of the animals through multiple targets. Moreover, the observed synergistic effects may not be limited to the treatment of sepsis, but also enhance the individual roles of each mechanism in a number of other bacterial infections, with a potential greater role in the field of infectious diseases.

Recently, Meng et al. (47) identified a novel type of mesenchymal-like stem cells, endometrial regenerative cells (ERCs), from menstrual blood. It was also reported that stromal cell-derived factor-1 (SDF-1)-pretreated ERCs exerted therapeutic effects by alleviating sepsis-related symptoms, reducing tissue damage, modulating inflammatory imbalance, and alleviating oxidative stress in a mouse model of sepsis. SDF-1, the ligand for CXCR4, plays an important role in the migration of mesenchymal stromal cells (48). The *in vitro* experiments demonstrated that there was a much higher CXCR4 expression on ERCs when they were cocultured with SDF-1. The *ex vivo* experiment results showed that SDF-1 expression significantly increased in mouse tissues. Furthermore, it was also found that SDF-1-pretreated ERCs contributed to reducing the levels of proinflammatory cytokines (TNF-α, IL-1β) and increasing the levels of anti-inflammatory factors (IL-4, IL10) in mouse serum, liver, and lung. Moreover, SDF-1-pretreated ERCs could also significantly decrease the levels of iNOS and MDA and increase the expression of Nrf2, HO-1, and SOD in liver tissues.

In summary, the combination of MenSC and antibiotics may improve sepsis survival through multiple targets and have a therapeutic effect on sepsis through pretreatment. In addition, MenSC have higher proliferative activity compared to both UCB-MSC and BMSC (49). Compared with other stem cells, the harvesting of MenSC has the advantages of easy and non-invasive collection, low immunogenicity, wide source of material, and no ethical controversy, which makes it highly probable that MenSC will replace the traditional BMSC and play an important role in the future treatment of clinical diseases (50).

## Summary and concluding remarks

**Figure 3:**
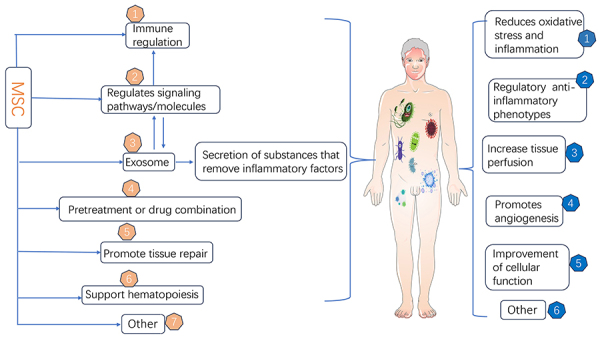
Mesenchymal stem cell (MSC) therapeutic pathway in sepsis. The therapeutic effects of MSC in sepsis involve multiple interacting mechanisms. Key areas of research are the regulation of signaling pathways/molecules associated with inflammation, immunomodulation, exosomes to secrete anti-inflammatory factors, and pretreatment of MSC or in combination with other treatments.

The therapeutic effects of MSC on sepsis involve multiple interacting mechanisms. Key areas of research involve use of MSC for regulation of signaling pathways/molecules associated with inflammation, immunomodulation, exosomes to secrete anti-inflammatory factors, and pretreatment with MSC or MSC in combination with other treatments (Figure 3). The pathophysiological features of sepsis, including the main aspects of inflammatory imbalance, immunosuppression and coagulation abnormalities, and the biological characteristics of MSC from different sources were combined in this article and are summarized in Table 1. For clinical application, industrialized *in vitro* culture is required.

**Table 1:** Characteristics of mesenchymal stem cells (MSC) from different sources.

Content of MSC from different sources	MSC proliferative capacity	Immunomodulatory capacity	Cytokines secreted by exosomes
ADSC>UCB-MSC, UC-MSC, BMSC, MenSC 26	MenSC>UCB-MSC, BMSC 49	UC-MSC, ADSC>BMSC>UCB-MSC 30 43	VEGF: BMSC>UC-MSC 20 G-CSF, GM-CSF, HGF, IL-6, IL-8, IL-11: UC-MSC>BMSC 36 Cytokines that support hematopoiesis: UCB>BMSC 44 (cytokine secretion of MSC from different sources have different characteristics)

ADSC: adipose derived stem cell; UCB-MSC: umbilical cord blood mesenchymal stem cells; UC-MSC: umbilical cord mesenchymal stem cells; BMSC: bone marrow mesenchymal stem cell; MenSc: menstrual blood derived mesenchymal stem cell.

Currently, there are few human trials on MSC treatment of sepsis, which is insufficient to guide the determination of clinical protocols. We summarized the data of the completed clinical trials (Supplementary Table S1), and there are only trials on the treatment of sepsis with BMSC, ADSC, and UC-MSC. The mode of administration was always intravenous infusion, and the doses ranged from 0.3×10^6^ to 3 ×10^7^ cells/kg. However, individual variability, therapeutic route, timing of treatment, selection of different sources of MSC and their dosage, duration of action, clinical safety, other therapeutic modalities of MSC in combination or pretreatment, and manufacturing process are also aspects that need to be taken into consideration. Hopefully, MSC from more sources will be involved in therapeutic trials for sepsis in the future.

While MSC have shown immunomodulatory potential, their feasibility and effectiveness for sepsis treatment remains to be determined. Sepsis progresses rapidly with a highly acute course, thus the role of MSC pretreatment needs further investigation. Moreover, sepsis patients have rapid disease progression, and MSC acquisition, isolation, and identification require time, so the stability of stored MSC is a current issue that needs to be further addressed. Basic studies on MSC cryopreservation have shown that freshly thawed MSC have diminished immunosuppressive capacity and reduced ability to inhibit T cell proliferation (51). This may have a significant impact on the therapeutic efficacy of MSC.

In addition, MSC are highly heterogeneous and there is no standardized production process. It has been shown that even small changes in isolation and culture protocols, such as centrifugation speed, medium composition, and serum concentration, can significantly affect the yield, quality, and composition of isolated cell populations, and differences in MSC isolation and purification may result in the display of different immunomodulatory properties (52). Finally, most clinical studies were double-blind or randomized controlled trials, and the difficulty of long-term follow-up after infusion prevents the observation of adverse reactions and sequelae. There are no clear rules for the selection of MSC from different sources, and there are also differences in the injection dose, injection method, administration time, and number of administrations. In the published results of clinical trials on MSC in the treatment of sepsis, although no serious adverse reactions have been reported, mild adverse reactions during the trials have occurred, including fever, transient facial flushing, throat irritation, itching, transient hypoxia, cardiac arrhythmias, and fungal infections (21,39,53).

In summary, MSC should be considered as potential adjuvant therapies, rather than a definitive treatment, for constraining sepsis progression. Their clinical effectiveness requires validation beyond experimental models. Further research needs to focus on addressing the barriers to clinical application of MSC as a sepsis therapy.

## Supplementary Material

Click to view [pdf].
