# Supplementary material for: Mesenchymal stem cells from different sources for sepsis treatment: prospects and limitations
Source: Braz J Med Biol Res. 2024 Oct 14;57:e13457. doi: 10.1590/1414-431X2024e13457 (PMC11484354; doi:10.1590/1414-431X2024e13457)
Supplement: Supplementary file 1 [file 1414-431X-bjmbr-57-e13457-suppl.pdf]

Supplementary Table S1. Trials of mesenchymal stem cells (MSC) in the treatment of sepsis.

MSC sources	Subjects	Results	Conclusion	MSC dose/choice of treatment	Reference	Study type
ADMSC	10 people	↓TNF-α, IFN-γ<br>↓SOFAscore (adjusted)<br>↑survival rate on the 14th day and 28th day.<br>↑Clinical improvements	MSC treatment may have a positive impact on the survival rates of sepsis during the early phase. However, further randomized controlled studies with a large group of patients are needed.	1×10^6/kg intravenous infusion	(31)	*In vivo*
ADMSC	macrophage	↓macrophage M1<br>↑macrophage M2<br>↓IL-1β, TNF-α, and IL-6	Exosomes from adipose-derived stem cells alleviate the inflammation and oxidative stress via regulating Nrf2/HO-1 axis in macrophages.	–	(27)	*in vitro*
ADMSC	mice	↓ IL-27, IL-6, TNF-α, and IL-1β,<br>↑survival rate	ADMSC-exosomes inhibited IL-27 secretion in macrophages and alleviated sepsis-induced ALI in mice.	–	(28)	*In vivo*
BMSC	9 people	There were no prespecified MSC infusion-associated or serious unexpected AEs, nor any safety or efficacy signals for the expected AEs or the measured cytokines between the interventional and observational cohorts.	The infusion of freshly cultured allogenic bone marrow-derived MSC, up to a dose of 3 million cells/kg (250 million cells), into participants with septic shock seems safe.	0.3, 1.0, and 3.0×10^6 cells/kg (250 million cells) intravenous infusion	(21)	*In vivo*
BMSC	rats	↓Endotoxin and inflammatory cytokines<br>↓Mortality, intestinal lesion	BMSC can eliminate endotoxemia and reduce mortality in rats with ALF, and the PI3K/AKT/mTOR signal pathway is involved in intestinal differentiation.	–	(15)	*In vivo*
BMSC	24 adult male albino rats	↑ Histopathological changes of the tissue<br>↑Regeneration liver<br>↓ Apoptosis	BMSC therapies could be a viable approach to treat sepsis-induced liver damage by activating Nrf2 signaling.	–	(16)	*In vivo*
BMSC	rats	↓Histopathological injury<br>↓ROS, MDA, SOD, GSH-Px, CAT<br>↓(TNF)-α, (IL)-1β, and IL-6<br>↑ Nrf2 , HO-1 and four-day survival rate	Nrf2-mediated HO-1 signaling pathway plays a critical role in the protective effects of BMSC on LPS-induced ALI.	–	(17)	*In vivo*
BMSC	mice	↑Tissue injury and mortality. MSC silenced for SDC2 had a decreased ability to promote phagocytosis of apoptotic neutrophils by macrophages in the peritoneum.	Mesenchymal stromal cell-derived syndecan-2 regulates the immune response during sepsis to foster bacterial clearance and resolution of inflammation.	–	(18)	*In vivo*
BMSC	Male mice	↓Lung W/D ratio, NF-KBp65 expression, and the levels of TNF-α, IL-1β, IL-6	*Vibrio vulnificus* sepsis can cause acute lung damage and edema, and BMSC can down regulate inflammatory cytokines, reduce lung injury caused by *Vibrio vulnificus* sepsis.	–	(19)	*In vivo*
UC-MSC	15 people	↓L-6,<br>↓IL-8,<br>↓TNF-α<br>↓CRP	A single intravenous infusion of allogeneic MSC up to a dose of 3×10^6 cells/kg was safe and well tolerated in 15 patients with severe sepsis.	(1×10^6 cells/kg), intermediate (2×10^6 cells/kg), and high (3×10^6 cells/kg) intravenous infusion	(37)	*In vivo*
UC-MSC	mice	↓(Cr), BUN<br>↑Survival rate	UC-MSC exosomes alleviate sepsis-associated acute kidney injury via regulating microRNA-146b expression.	–	(34)	*In vivo*
UC-MSC	mice	↓IL-6, IL-1β, TNF-α, HMGB1, NF-κB	Therapeutic effects of UC-MSC on sepsis-associated encephalopathy in mice by regulating PI3K/AKT pathway.	–	(35)	*In vivo*
UC-MSC	17 people	↓Levels of ferritin, IL-6 and MCP1-CCL2<br>↓Reactive C-protein, D-dimer and neutrophils<br>↑TCD3, TCD4 and NK lymphocytes	UC-MSC infusion is safe and can play an important role as an adjunctive therapy both in the early stages, preventing severe complications, and in the chronic phase with post-acute sequelae reduction in critically ill COVID-19 patients.	Three doses of 5×10^5 cells/kg UC-MSC	(38)	*In vivo*
UC-MSC	18 people	No serious UC-MSC infusion-associated adverse events were observed	Intravenous UC-MSC infusion in patients with moderate and severe COVID-19 is safe and well tolerated.	Intravenous infusion of UC-MSC (3×10^7 cells per infusion)	(39)	*In vivo*
UCB-MSC	mice	↑IL-10<br>↓Mortality, recruitment of neutrophils to the liver	hUCB-MSC have beneficial effects against LPS-induced sepsis through associations with neutrophils.	–	(42)	*In vivo*
MenSC	mouse	↑Survival rate<br>↑Bacterial clearance in the peritoneal fluids and blood<br>↓Pro- and anti-inflammatory cytokines	MenSC in combination with antibiotics enhance survival in CLP-induced sepsis by acting on multiples targets.	–	(46)	*In vivo*

MenSC	mouse	↓TNF-α, IL-1β<br>↑IL-4, IL10<br>↓iNOS, MDA<br>↑Nrf2, HO-1, and SOD	SDF-1 pretreatment plays a key role in improving the therapeutic effects of ERCs in alleviating sepsis-related symptoms, reducing tissue damage, regulating inflammatory imbalance, and relieving oxidative stress in a mouse sepsis model.	–	(48)	*In vivo*

G-CSF: granulocyte colony-stimulating factor; HGF: human growth factor. IL: interleukin; AE: adverse effects; BMSC: bone marrow mesenchymal stem cells; ADSC: adipose stem cells; UC-MSC: umbilical cord mesenchymal stem cells; UCB-MSC: umbilical cord blood mesenchymal stem cells; MenSC: menstrual blood mesenchymal stem cells.
